# Synthesis and characterization of a functional benzoylecgonine – bovine serum albumin conjugate for quantification of a humanized monoclonal anti-cocaine antibody

**DOI:** 10.1016/j.bbrep.2025.102282

**Published:** 2025-09-27

**Authors:** Rose P. Webster, Terence L. Kirley, Andrew R. Ray, Zhen-Hin Chan, Andrew B. Norman

**Affiliations:** Department of Pharmacology, Physiology, and Neurobiology, University of Cincinnati College of Medicine, Cincinnati, OH, 45267-0575, USA

**Keywords:** BE-diab BSA, Cocaine, Anti-cocaine antibody, Humanized, Monoclonal, ELISA, Antigen-synthesis

## Abstract

We have developed a humanized monoclonal anti-cocaine antibody (h2E2), as a potential solution for cocaine use disorder. A key milestone was the development of an assay to quantify this monoclonal antibody (mAb) in animal and human blood. Thus, we synthesized a novel benzoylecgonine-1,4-diaminobutane-BSA (BE-diab BSA) conjugate as an antigen for quantifying h2E2 using ELISA. We report here the method of synthesis of this conjugate, BE-diab BSA, and assessment of its binding to the h2E2 mAb using an ELISA and a fluorescence quenching assay. Compared with four commercial BE-BSA conjugates, BE-diab BSA demonstrated markedly stronger mAb binding in ELISA—three of the commercial conjugates showed less than 10 % relative binding. Fluorescence quenching assays confirmed this binding superiority, with the commercial conjugates showing minimal mAb interaction, while BE-diab BSA induced robust intrinsic fluorescence quenching. SDS-PAGE analyses identified structural differences consistent with binding results between our functional conjugate and the commercial preparations. This functional and reproducible in-house conjugation has been integrated into a GLP-validated ELISA, which is now in use for pharmacokinetic analyses and for qualifying antibody release lots for clinical deployment.

## Highlights

• **Several commercial BE-BSA conjugates were found to be unsuitable for ELISA h2E2 mAb quantitation**.
• **A novel, spacer-linked benzoylecgonine-BSA conjugate synthesized and characterized**.
• **Rapid and simple fluorescence quenching assay developed to test the BE-BSA conjugate**.
• **Successful and reproducible ELISA quantitation of h2E2 mAb using the BE-BSA conjugate**.
• **External GLP lab used the conjugate successfully in h2E2 anti-cocaine mAb release assay**.

## Graphical abstract

**Figure:**
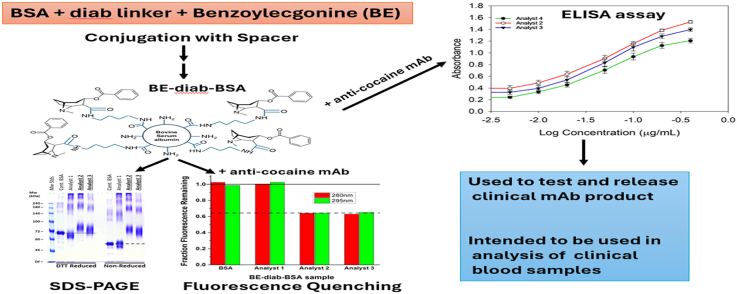

## 1 Introduction

To enable clinical trials of our h2E2 anti-cocaine mAb as a treatment for cocaine use disorders, several FDA mandated milestones must be achieved. An essential prerequisite for pharmacokinetic studies in clinical trials was to quantify the mAb by its binding either to cocaine itself or a structurally related molecule such as benzoylecgonine (BE). BE-diab BSA is a BSA conjugate of BE, with a short linker between BSA and BE. BE, although a pharmacologically inactive metabolic product of cocaine, binds with high affinity to h2E2. BE was coupled to BSA by carbodiimide activation of its carboxyl group by employing a four-carbon diamine spacer. Use of this specific BE-diab BSA conjugate instead of a species-specific capture antibody to quantify h2E2 mAb in blood minimized matrix interference issues with human blood matrices and meets FDA assay requirements.

In the absence of an FDA approved pharmacotherapy for cocaine use disorder and the failure of dopamine antagonists or agonists to prevent cocaine abuse, the h2E2 mAb offers a promising alternative [1]. h2E2 binds cocaine with high affinity, blocks brain entry in animal models [2,3] and exhibited favorable pharmacokinetics, including a low volume of distribution and a long elimination half-life [2]. Due to the absence of suitable cocaine and cocaine metabolite commercial conjugates, the reproducible synthesis and validation of BE-diab BSA described herein is a critical advancement in the clinical development of h2E2.

## 2 Materials and methods

### 2.1 Materials

For antigen synthesis, bovine serum albumin (BSA; A4378), 2-(N-morpholine) methane sulfonic acid (MES), 1,4-diaminobutane (D13208), N-hydroxylamine hydrochloride (159417), and benzoylecgonine tetrahydrate (B4147) were purchased from Sigma Aldrich (St. Louis, MO). Centricon 10,000 MW cutoff filters were purchased from Millipore Sigma. 1-ethyl-3-[dimethylaminopropyl]carbodiimide (EDC) (PG82079), N-hydroxysuccinimide (PG82071), and dialysis tubing were purchased from Thermo Fisher Scientific. Commercial BE-BSA antigens and crossmatched antibodies were purchased from Fitzgerald (two conjugates 801037 and 81B29, and two antibodies 101370 and 10B11G), My Biosource (MBS3033688, MBS5305238) and BiosPacific (V5200050, A52025501P).

SDS-PAGE gels, including acrylamide, bis-acrylamide and Coomassie blue were from BioRad. Pre-stained molecular weight standards (10–240 kDa) were purchased from SMOBIO Technology, Inc.

h2E2 was manufactured by Catalent PharmaSolutions (Madison, WI).The secondary ELISA antibody biotinylated goat anti-human IgG (GAH), and alkaline phosphatase substrate (para-nitro phenyl phosphate (pNPP)), were from Sigma Aldrich (St. Louis, MO). Streptavidin linked-alkaline phosphatase was acquired from Roche (Indianapolis, IN).

### 2.2 Antigen synthesis

**Fig. 1:**
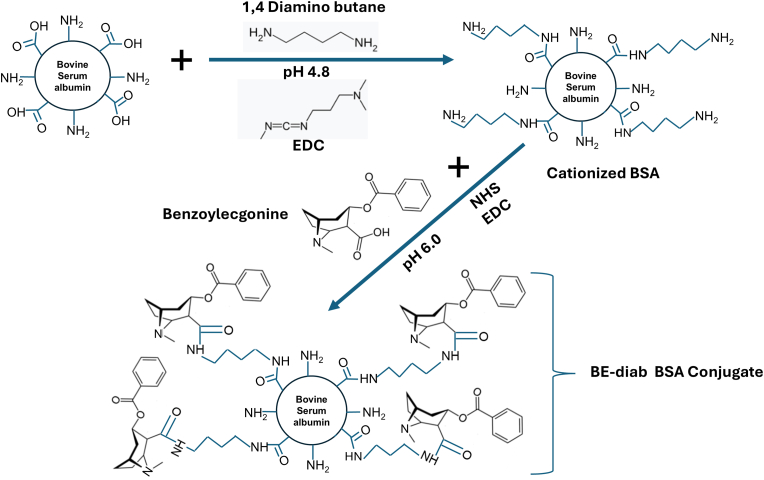
Synthetic strategy used for the synthesis of BE-diab BSA conjugate. BSA is reacted with the spacer 1,4 diamino butane in the presence of EDC in acidic conditions. The resulting BSA-spacer linked via an amide linkage is then reacted with BE resulting in the BE-BSA conjugate, wherein the carboxyl group of BE is in an amide linkage with the amino group of the spacer (see text for complete details).

BSA was first dialyzed against water (4h, at 4 °C) and then overnight at 4 °C against 0.1 M 2-(N-morpholino) ethanesulfonic acid (MES), 154 mM NaCl, pH 4.8, to remove any low molecular weight impurities. BSA was cationized (increased in net positive charge) to facilitate subsequent conjugation by crosslinking with the spacer and then conjugated to benzoylecgonine (see Fig. 1). After dialysis, BSA was collected, and its concentration determined by absorbance at 280 nm (extinction coefficient = 0.667 for 0.1 % solution of BSA) before reacting with 1,4-diaminobutane. This BSA (5 mL of ∼10 mg/mL) was reacted with 1,4-diaminobutane (957 μl) in the presence of EDC (at 2 mg/mL) at a strictly controlled pH of 4.8. The pH of 1,4-diaminobutane solution was adjusted down to pH 4.8, first with 6 N HCl and then with 1 N HCl, with constant stirring on a magnetic stir plate and monitoring using a pH meter. The dialyzed BSA (7–10 mg/mL) was added to this solution with constant stirring, and the pH monitored and maintained at pH 4.8 with 0.1 N HCl. After approximately 5 mL of the BSA was added, EDC was added (to a final concentration of 2 mg/mL) and allowed to mix for 2 h at 22 °C with continuous pH monitoring and HCL additions to maintain the reaction at pH 4.8. After 2 h, an additional amount of EDC (at a concentration of 1 mg/mL) was added and allowed to mix for another 2 h at a controlled pH of 4.8. The reaction was stopped by adding hydroxylamine to a final concentration of 30 mM, and then all reagents were removed by dialysis at 4 °C, first against water and then with 2 rounds of dialysis against 0.1 M MES, 154 mM NaCl, pH 6.0. Following overnight dialysis, this cationized BSA was concentrated to 7–10 mg/mL with Centricon 10k MWCO concentrators. All of this (∼30 mg) 7–10 mg/mL cationized BSA was then reacted with 30 mg of benzoylecgonine in the presence of EDC (118 mg) and NHS (24 mg) in a final total volume of 5 mL for 4 h at 22 °C. The conjugation reaction was stopped with addition of a final concentration of 76 mM N-hydroxylamine-HCl. The conjugated product was dialyzed at 4 °C twice (overnight and then 4 h the next day) versus distilled water. The concentration of the synthesized BE-diab BSA conjugate product was determined using the absorbance at 280 nm. We then tested our BE-diab BSA conjugate and several commercial conjugate preparations of BE-BSA for h2E2 mAb binding using ELISA and fluorescence quenching assays. All conjugates were also examined for protein structural and oligomeric differences using reducing and non-reducing SDS polyacrylamide gels.

### 2.3 Fluorescence quenching assay

Fluorescence quenching measurements of various BE-diab BSA conjugates, and by BSA alone, were made using a 2.0 mL sample in a 1 cm square fluorescence cuvette, in a Hitachi F-2000 fluorescence spectrophotometer at 20 °C. Initial fluorescence of 20 nM h2E2 mAb was measured in tris buffered saline, pH = 7.4, reading fluorescence emission at 330 nm after excitation at either 280 nm or 295 nm. The decrease in fluorescence due to quenching of the tyrosine and tryptophan mAb fluorescence by the BE-diab BSA conjugate was measured after addition of a final concentration of 4 nM of different preparations of BE-BSA or control BSA. A single saturating concentration was used in this assay to rapidly screen useful conjugates. This simple assay rapidly identified conjugates likely to bind to the mAb most effectively (i.e. quenching the mAb intrinsic fluorescence as does unconjugated BE [4]).

### 2.4 ELISA

ELISA was performed using a modification of previously published methods [5]. Briefly, 2 μg/mL of the BE-BSA or BE-diab BSA conjugates were adsorbed onto the 96 well plates for 1 h, and these plate wells were blocked using BSA-Tris buffer containing 2.5 % BSA (10 mM Tris, 140 mM NaCl, and 0.02 % NaN_3_, pH 7.2) for 15 min. After removal of the blocking solution, the h2E2 mAb was then captured by binding to the benzoylecgonine 1,4-diaminobutane-BSA (BE-diab BSA) conjugates on the plate for 1 h. After removal of the samples and buffer washing, the captured h2E2 in the plate wells was detected by incubation with the secondary antibody, a biotinylated goat anti-human Fc specific antibody. All washes following the h2E2 incubation were performed with BSA-PBS buffer (0.5 % BSA, 10 mM sodium phosphate, 145 mM NaCl, 1.5 mM MgCl_2_, 0.05 % Triton X-100, and 0.02 % NaN_3_, pH 7.2). The colorimetric signal was developed after addition of a streptavidin-alkaline phosphatase conjugate and its substrate, 4-nitrophenyl phosphate, and the resulting 405 nm absorbance was read (for details see Refs. [2,6,7]. All samples were assayed in triplicate. Antigen binding curves were generated using a SpectraMax M3 Multimode Microplate reader and SoftMax Pro software was used to generate a 4-parameter logistic fit to the ELISA data. All statistical analyses of the ELISA data generated on the SpectraMax plate reader were carried out using Sigma Plot.

### 2.5 SDS-PAGE electrophoresis

7 % SDS-PAGE gels were poured, run, and stained with Coomassie Blue, basically as published previously [8]. Protein samples (0.3 mg/mL) were prepared in either non-reducing sample buffer or reducing sample buffer (containing 100 mM DTT), also containing 5 % SDS, and 25 % glycerol. Samples were boiled for 5 min, each lane (except lane one) was loaded with 3 μg sample and run on 7 % acrylamide gels. Pre-stained molecular weight standards (10 μL) were loaded in lane one. All images showing comparisons of samples, conditions and effects on electrophoretic mobility were generated from the same gel. Electrophoresis was terminated when the bromophenol blue tracking dye approached the bottom of the gel. Gels were incubated at room temperature for 1 h with Coomassie blue stain, and then destained for 4–20 h before being photographed. Each electrophoresis gel image depicts a single SDS-PAGE, allowing direct comparisons of the protein bands.

## 3 Results

**Fig. 2:**
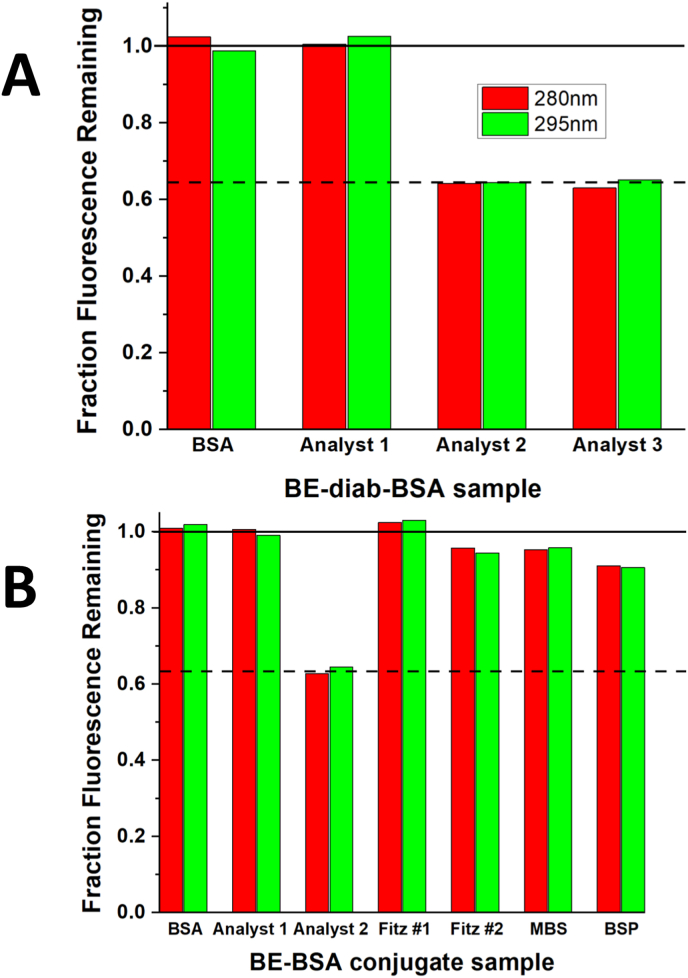
Fluorescence quenching of h2E2 mAb by BE-BSA conjugates. **Panel A:** Fluorescence emission at 330 nm (excitation at 280 nm for tyrosine and tryptophan or 295 nm for tryptophan only) shows h2E2-induced quenching for functional in-house conjugates (analysts 2 and 3) but not analyst 1, who used a different, non-functional synthetic method. **Panel B:** Comparison of analyst 1 and 2 conjugates with four commercial BE-BSA preparations: Fitzgerald Cat# 801037 (Fitz#1), Fitzgerald Cat# 801B29 (Fitz#2), My Biosource (MBS3033688), and BiosPacific (BSP V52000501). All commercial conjugates showed minimal quenching, with BSP demonstrating partial activity (∼30 %).

**Fig. 3:**
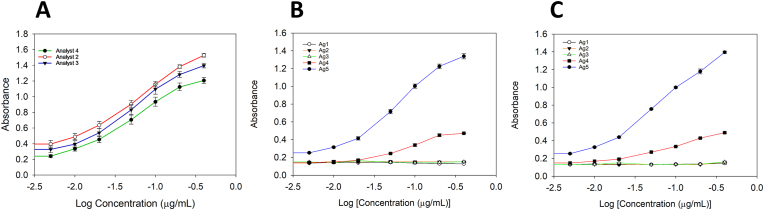
ELISA evaluation of h2E2 mAb binding to BE-BSA conjugates All figures are a plot of absorbance vs log of the concentration of the antibody h2E2 tested against 96 well plates coated using 2 μg/mL of BE-diab BSA synthesized in our laboratory by 3 different analysts (2, 3 and 4), using the synthetic protocol described in this study. **Panel A**: each product was tested on 3 separate days in assays using triplicates each day (n = 9). All antigens were tested against a range of h2E2 mAb concentrations (0–0.4 μg/mL). **Panel B**: Binding comparison between in-house antigen (Ag5) and commercial conjugates—Fitz#1 (Ag1), Fitz#2 (Ag2), MBS (Ag3), BSP (Ag4). All antigens were tested under identical conditions against h2E2 at a concentration range of 0–0.4 μg/mL. Commercial antigens were tested once (n = 3); for the in-house antigens a mean of absorbance obtained at each concentration of the antibody (h2E2) using analyst 3 (n = 3) or analyst 4 (n = 3) conjugates assayed on two separate days. **Panel C**: Antigens tested were the in-house synthesis (Ag5), Fitzgerald antigen 801037 (Ag1, FITZ#1), Fitzgerald antigen 801b29 (Ag2, FITZ#2), My Biosource antigen (Ag3, MBS) and BiosPacific antigen (Ag4, BPS). All commercial antigens were tested at a concentration of 2 μg/mL against vendor supplied antibody at a concentration range of 0–0.4 μg/mL. The in-house synthesized antigen (Ag5, mean absorbances of analyst 3 and 4, n = 3 for each) was tested against h2E2 (concentration 0–0.4 μg/ml).

**Fig. 4:**
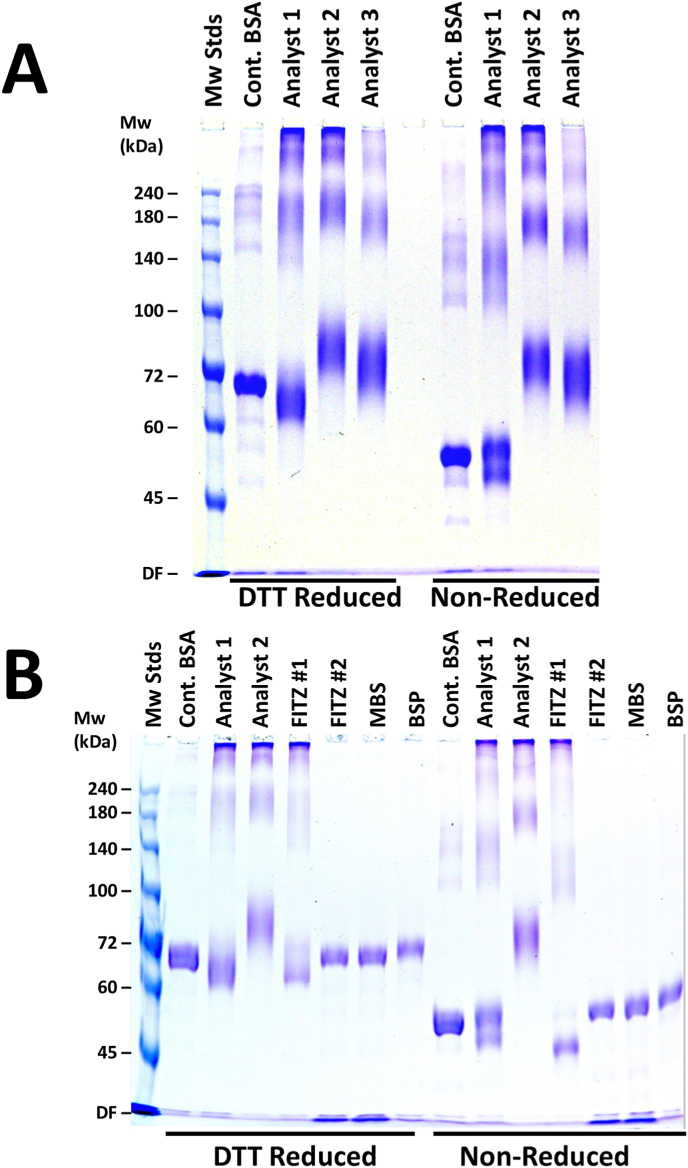
SDS-PAGE analyses of various BE-BSA conjugates. **Panel A:** Reducing and non-reducing SDS-PAGE (7 % gel) of three in-house conjugates (3 μg/well). Functional conjugates from Analysts 2 and 3 show distinct migration compared to non-functional conjugate from Analyst 1, made using an alternative method. **Panel B:** Comparative SDS-PAGE of in-house conjugates (Analyst 1 and 2) and commercial BE-BSA products (Fitz#1, Fitz#2, MBS, BSP), all analyzed under both reducing and non-reducing conditions at 3 μg/well. Analyst 1 synthesis (a previous, undescribed conjugation synthetic strategy that generated a non-functional conjugate) differs from analysts 2 and 3, who synthesized antigen using the methods described in this work. Differences in band migration correlated with functional assay results.

Fig. 1 shows the synthetic scheme used to generate our in-house BE-BSA conjugate. We compared our in-house and several commercial BE-BSA conjugates using an intrinsic mAb fluorescence quenching assay that was developed as a predictive screening assay that could quickly indicate which BE-BSA conjugates are likely to work in the ELISA (Fig. 2). We also then confirmed that our in-house synthesized conjugates did work in the ELISA (Fig. 3) and carried out SDS-PAGE (Fig. 4) to examine the possible molecular differences that could have contributed to the success/failure of these conjugates to bind to the h2E2 mAb in the ELISA and fluorescence quenching assays.

### 3.1 Fluorescence quenching assay

Fig. 2 Panel A shows a comparison of different conjugates prepared by different synthetic protocols used in our laboratory. In this assay, the decrease in fluorescence is due to quenching of intrinsic mAb tyrosine and tryptophan fluorescence by the mAb bound BE in the BE-BSA conjugates. A substantial decrease in mAb fluorescence indicates high affinity binding of the conjugate to the mAb. Thus, conjugates having no BE accessible for binding to mAb lead to no quenching; whereas successful BE conjugates result in maximal quenching of mAb fluorescence. Conjugates synthesized in-house using the optimized protocol by analysts 2 and 3 showed substantial fluorescence quenching, indicating effective mAb binding. Analyst 1's conjugate, made with a previously unsuccessful method, showed minimal quenching and served as a negative control. This quenching behavior is seen very clearly in the conjugates prepared in house by analysts 2 and 3 in Fig. 2, Panel A. Fig. 2, panel B shows that none of the 4 commercial conjugates (FITZ #1, FITZ #2, MBS, and BSP) decreased the mAb fluorescence to a similar extent as did one of our successful BE-diab BSA conjugate syntheses (analysts 2 and 3), i.e., they did not bind efficiently to the BE/cocaine binding site on the mAb. BiosPacific's conjugate (BSP) exhibits some binding in ELISA (see Fig. 3B) and also is the most effective amongst commercial conjugates in this fluorescence quenching assay (the “BSP” bars at the far right of Fig. 2, panel B), although far less (approximately 30 %) than the quenching efficiency of the in-house synthesized BE-diab BSA antigen. In both panels of Fig. 2, unmodified BSA was used as a negative control, which showed no quenching, as expected.

### 3.2 ELISA assay

In-house conjugates synthesized by analysts 2–4 produced similar ELISA binding curves across three experimental days as evidenced by the ELISA data shown in Fig. 3, Panel A. ELISA was performed over 3 different days with triplicates at each concentration of conjugated antigen in all experiments. Two-way ANOVA showed no overall differences (p = 0.627). An F-test was carried out to examine dose-response similarities or parallelism. It was observed that although there was a significant difference in the dose-response curves between analyst 4 and analyst 2 (p = 0.0015), there was no significant difference between analyst 2 and analyst 3 (p = 0.147). The 2-way ANOVA identifies further that the differences in data generated using the conjugates synthesized by analyst 2 and 4 was at the highest 2 doses of antibody tested (0.4 and 0.2 μg/mL). Analyst 4's conjugate was not evaluated in fluorescence quenching or SDS-PAGE. Fig. 3, Panel B shows that commercial BE-BSA conjugates showed negligible binding to h2E2 by ELISA, except BSP, which reached ∼29 % (see the red curve) of the in-house conjugate's response at the highest mAb concentration (blue curve). Fig. 3, Panel C shows a comparison of our in-house conjugate (with h2E2) to the commercial ones, using their corresponding matched antibodies. This confirmed the very limited utility of commercial conjugates for detecting and quantitating h2E2.

### 3.3 SDS-PAGE analyses of conjugates

Electrophoretic profiles of BE-BSA conjugates were compared under reducing and non-reducing conditions to assess molecular differences influencing mAb binding. Fig. 4 shows a comparison of the electrophoretic migration patterns of our laboratory generated BE-diab BSA antigens (panel A) and the commercial BE-BSA antigens (panel B) using both reduced and non-reduced conditions. As seen in Panel A, on reduction, the dominant stained protein band was slightly above 72 kDa for both analyst 2 and 3 syntheses (functional conjugates) for both reduced and non-reduced samples. The dominant stained protein band resulting from analyst 1 synthesis (which is a non-functional conjugate) was observed at lower than 72 kDa under non-reducing conditions, more similar to the control BSA and to all 4 of the (non-functional) commercial antigens tested (Panel B). The conjugate protein band patterns were very similar for analyst 2 and 3 under both reduced and non-reduced conditions, in contrast to the differences seen upon reduction for the analyst 1 sample and all four of the commercially prepared BE-BSA conjugate samples.

We note that analyst 1 synthesis followed a different synthetic protocol than that described in this study, which was not successful, since the resulting BE-BSA conjugate did not show binding to the h2E2 mAb, as seen by a lack of mAb fluorescence quenching (Fig. 2) and no measurable signal in ELISA (unpublished data). Thus, analyst 1 conjugate served as a negative control for our in-house syntheses. It performed similarly to the 4 commercial BE-BSA antigens in all assays performed. Three of the 4 commercially available conjugates (all but the FITZ#1 conjugate) showed banding patterns virtually identical to unmodified BSA under both reducing and non-reducing SDS-PAGE conditions (Fig. 4, panel B).

## 4 Discussion

Traditionally, a small molecule antigen is coupled to a larger protein such as BSA to increase its immunogenicity [9]. In our laboratory, benzoylecgonine was attached to BSA to measure the amount of h2E2 in blood, plasma, or serum after it was administered to rats or mice [2,3]. Benzoylecgonine (BE) was used for this purpose instead of cocaine, since BE has a free carboxylic acid group that can be activated to enable chemical conjugation, unlike cocaine which contains a carboxyl methyl ester at this site. It is important to recognize that the mAb developed in response to the BE-generated antigen binds cocaine with higher affinity (4 nM Kd) than it binds BE (20 nM Kd), as documented in several published studies using several different binding methods by our laboratory [[2], [3], [4],[10], [11], [12]]. Typically, conjugation methods used for this purpose include carbodiimide and maleimide based methods [13]. In this case, carbodiimide conjugation involves activation of BSA carboxylic acids by the water-soluble EDC reagent, which is then used for direct linkage with primary amines on a 4-carbon spacer/linker, forming amide bonds. Utilizing a spacer between the small molecule and the carrier protein has been a longstanding strategy to improve the immunogenicity of small molecules under 1000 Da [[14], [15], [16], [17], [18]]. This same strategy is also employed to increase the affinity of the antibody to the plate-coated BE-diab BSA conjugate. Since proteins contain both amines and carboxylic acids, EDC mediated conjugation often can cause random polymerization of proteins. This can result in a high concentration of the immunogenic small molecules to be concentrated at high densities on some carrier proteins such as BSA or keyhole limpet hemocyanin (KLH). In the synthetic protocol described in this study, we used a 4-carbon diamine linker which has been shown in other studies to increase the immunogenicity of the synthesized BE-BSA-conjugate [14,19]. A spacer arm for the conjugate works to distance the characteristic structure of the antigen target, i.e., BE, away from the carrier protein, thereby increasing its exposure to the antibody. A medium length (3–5) spacer arm like that used here has been shown to prevent the shielding effects of the carrier proteins towards the target epitopes [20].

We demonstrated that the BE-diab BSA conjugate synthesized in our laboratory using the described synthetic protocol quenches mAb intrinsic fluorescence, just as BE and cocaine do [4] (see Fig. 2), and is suitable for the ELISA assay to quantify h2E2 mAb (Fig. 3). We have used this antigen and quantified h2E2 (as well as its parent molecule 2E2) from blood of both rats [3,21] and mice [2]. This BE-diab BSA conjugate was also used to quantify this mAb in the bulk drug substance manufactured by our partnering GLP/GMP laboratory as well as in stability assays conducted by them over a period of 2 years, which demonstrates the stability of both the mAb and the synthesized BE-diab BSA conjugate (unpublished data). The same BE-diab BSA conjugate was also used as a positive control in immunohistochemistry studies that demonstrated the lack of tissue reactivity of the monoclonal antibody to a large panel of human tissues [10]. The GLP laboratory was able to successfully release the drug substance based on these studies utilizing our BE-diab BSA conjugate.

It is unclear why the commercially available BE-BSA conjugates were not functional in our ELISA and fluorescence quenching assays. Future studies could be designed to pinpoint the reasons for failure of some of the conjugates. One of the possible reasons could be the chemistry of the linkage, since the commercial vendors most likely did not use a 4-carbon spacer in the BE to BSA linkage, as we did. Our observations of the migration of these conjugates on reduced and non-reduced SDS-PAGE gels also suggest that there were differing amounts of both intramolecular and intermolecular cross-linking of BSA occurring during these different conjugate syntheses (Fig. 4). From the non-reducing SDS-PAGE results, the main protein band in the control BSA and all the non-functional BE-BSA conjugates has a higher electrophoretic mobility than all of the functional conjugates, suggesting that the non-functional conjugates have a more compact, non-reduced structure, perhaps because they have maintained more of the 17 native disulfide bonds in the BSA molecule intact, which would lead to higher electrophoretic mobility due to a more compact structure after SDS denaturation. In addition, it is possible that these commercial conjugates had less than optimum BE to BSA labeling density (the commercial conjugates did not report BE to BSA stoichiometric coupling ratios, and we were unable to accurately determine that ratio on our own conjugates, due to the UV spectral overlap of the BE molecule with the BSA protein). However, it is possible to measure BE-BSA stoichiometry using either MALDI-TOF or using a chemical assay with 2,4,6-trinitrobenzenesulfonic acid (TNBS) [22,23]. Importantly, synthesis of the four-carbon spacer linked BE-diab BSA conjugate has played a critical role in our own studies of the pharmacokinetics of a h2E2 and has also enabled release of the manufactured h2E2 antibody lot by our GLP partners.

## CRediT authorship contribution statement

**Rose P. Webster:** Writing – review & editing, Writing – original draft, Supervision, Methodology, Investigation, Formal analysis, Data curation, Conceptualization. **Terence L. Kirley:** Writing – review & editing, Writing – original draft, Methodology, Investigation, Formal analysis, Conceptualization. **Andrew R. Ray:** Writing – review & editing, Investigation, Data curation. **Zhen-Hin Chan:** Writing – review & editing, Investigation, Formal analysis, Data curation. **Andrew B. Norman:** Writing – review & editing, Supervision, Resources, Project administration, Funding acquisition.

## Funding source

This work was supported by the United States National Institute on Drug Abuse grant U01DA050330.

## Declaration of competing interest

The authors declare the following financial interests/personal relationships which may be considered as potential competing interests:Andrew B. Norman reports financial support was provided by 10.13039/100000026National Institute on Drug Abuse. Andrew B. Norman has patent #10501556 issued to University of Cincinnati, E. R. Squibb & Sons, L.L.C. Andrew B. Norman has patent #9957332 issued to University of Cincinnati. Andrew B. Norman has patent #9758593 issued to University of Cincinnati, E. R. Squibb & Sons, L.L.C.

## Data Availability

Data will be made available on request.
